# Understanding the emotions of patients with inadequate response to antidepressant treatments: results of an international online survey in patients with major depressive disorder

**DOI:** 10.1186/s12888-018-1625-y

**Published:** 2018-02-05

**Authors:** Rajnish Mago, Andrea Fagiolini, Emmanuelle Weiller, Catherine Weiss

**Affiliations:** 1 Simple and Practical Mental Health, Philadelphia, PA USA; 2 University of Siena Medical Center, Siena, Italy; 3 H. Lundbeck A/S, Valby, Denmark; 4 Otsuka Pharmaceutical Development & Commercialization, Inc., Princeton, NJ USA

**Keywords:** Major depressive disorder, Inadequate response to ADTs, Frustration, Patient survey

## Abstract

**Background:**

Evidence suggests that nearly half of patients with major depressive disorder (MDD) do not achieve an adequate response to antidepressant treatments (ADTs), which impacts patients’ functioning, quality of life (QoL), and well-being. This patient survey aimed to better understand patient perspectives on the emotional impact of experiencing an inadequate response to ADTs.

**Methods:**

An online survey was conducted in 6 countries with respondents diagnosed with MDD and experiencing an inadequate response to ADTs. The survey was designed to explore how patients felt about their medications and health care provider (HCP). Those indicating they were ‘frustrated’ with their medications and/or HCP were asked to provide reasons for their frustration and its impact on their relationship with their HCP and decisions about their treatment.

**Results:**

Overall, 2096 respondents with MDD and inadequate response to ADT completed the survey. The most frequent emotion reported by patients regarding their medication was frustration (29.8% of respondents) followed by hopeless (27.4%) and apprehensive/anxious/scared (27.4%). Regarding their HCP, patients reported feeling understood (31.6%) and trusting/confident (28.8%) most often; however, 19.2% reported feelings of frustration. Main reasons for frustration with medication were poor symptom control/lack of efficacy (59.3%) and tolerability issues (19.7%), and the main reasons for frustration with their HCP were not feeling heard (22.4%), ineffective treatment (13.5%) and feeling rushed/lack of quality visit (12.5%). The longer the current episode duration and the greater the disruption to daily living, the more likely the respondents experienced feelings of frustration with medication. Feelings of frustration lead to adherence issues, with 33.3 and 27.3% of respondents indicating their frustration with their medication and HCP, respectively, made them want to quit their medication. Approximately one in six patients frustrated with either their medication and/or HCP indicated their frustration had resulted in them not taking their medication regularly. Frustration with their HCP also impacted patient’s confidence in HCPs abilities (34.7%), sharing less information with their HCP (28.9%) as well as missing appointments (17.4%) and medications (14.5%).

**Conclusions:**

Feelings of frustration are frequent in patients with inadequate response to ADT and this frustration may impact treatment adherence and the patient-HCP relationship.

**Electronic supplementary material:**

The online version of this article (10.1186/s12888-018-1625-y) contains supplementary material, which is available to authorized users.

## Background

The treatment of major depressive disorder (MDD) has progressed over the past several decades with greater awareness and understanding of the disease, better diagnosis, and medications with improved tolerability. However, despite the treatment advances and availability of multiple antidepressant classes/medications, approximately 50% of patients with MDD do not achieve an adequate response to antidepressant treatment (ADT) [1–3], as defined by a failure to achieve a response (50% or greater reduction in the severity of the depression) after treatment with an antidepressant at an adequate dose and duration (i.e., at least 6–8 weeks) [4]. From the patient perspective, people with MDD want their treatment to help them return to their usual level of functioning, return to their ‘usual self’ and to regain optimism and self-confidence [5].

Patients with inadequate response to ADT experience significant impairments in social functioning and often fail to regain a normal quality of life [6]. Work productivity loss of those employed and higher unemployment, along with higher emergency room utilization and hospitalization are also associated with an inadequate response to ADT [7]. Timely and effective treatment is important to prevent the long-term consequences of prolonged inadequately-treated MDD [2].

While the personal burden of an inadequate response can be significant and wide-ranging [8], little is known on the emotional impact of inadequate response to ADT. This survey set out to better understand the patient perspective on the emotional impact of experiencing an inadequate response to ADTs.

Specifically, the survey was designed to assess how frequently different emotions were reported in people with MDD experiencing an inadequate response to ADT, including the frequency of feelings of frustration with medication and their HCP. For those respondents who reported feeling frustrated with their medication and/or HCP, we sought to understand the reasons for their frustration and the impact this frustration had on their behavior.

## Methods

### Survey design and respondent criteria

This survey was conducted in the United States, Canada, UK, Germany, France, and Spain between 15 March and 16 June, 2016. Respondents were recruited using consumer panels where all panelists have provided consent to participate in research. An online questionnaire was used to screen respondents and collect quantitative data. Access to the survey was secure, ensuring the anonymity of respondents.

All respondents were eligible for the survey if they met the following criteria: age 18 to 65 years, diagnosed with depression by a healthcare professional; no comorbid diagnoses of bipolar disorder, schizoaffective disorder, or schizophrenia; experiencing symptoms of depression most of the time over the past week; their depression has a moderate to extreme impact on overall functioning as measured by the Sheehan Disability Scale (SDS) [9]; has a psychiatrist or primary care physician (or, in the US, a nurse practitioner or physician assistant) who is primarily responsible for their treatment; currently on an ADT for at least 6 weeks at an adequate dose (see Appendix); they felt that their depression has not improved or only a little since the onset of the current episode (as measured by the Patient Global Impression of Improvement (PGI-I)*,* and, finally, not working for a biasing employer (advertising agency or marketing research company or a company that manufactures, distributes or sells pharmaceuticals or health care products). Finally, per pharmacovigilance compliance regulations, all eligible respondents were required to provide consent for disclosure of any adverse-events reported within their responses.

### Defining an “inadequate response” to ADT

Eligible survey participants all suffered from MDD with a current episode having moderate to extreme impact on overall functioning as measured by SDS, and to be on at least one ADT at an adequate dose for at least 6 weeks. Participants were considered as having an inadequate response to their current ADT if they indicated that their current symptoms had little to no improvement (based on the 7-item PGI-C, ranging from very much worse to very much improved).

### Assessments

The survey was conducted as an online 20-min questionnaire. All data collected was self-reported by participants and included both close-ended and open questions on the following topics: history of depression (onset of first episode, number of lifetime episodes, length of current episode), treatment history (hospitalizations, psychotherapy, treatment received for current episode, reason for discontinuation); change on current treatment (patients’ global impression of change, PGI-C), current symptoms of depression, other current symptoms, impact of depression on functioning (SDS), treatment goals, emotional feelings about their experience with medication, healthcare providers and overall healthcare system, including feelings of frustration, intensity of frustration and reasons for feeling frustrated. Here we present the results pertaining to their feeling towards medication and HCP.

**Fig. 1:**
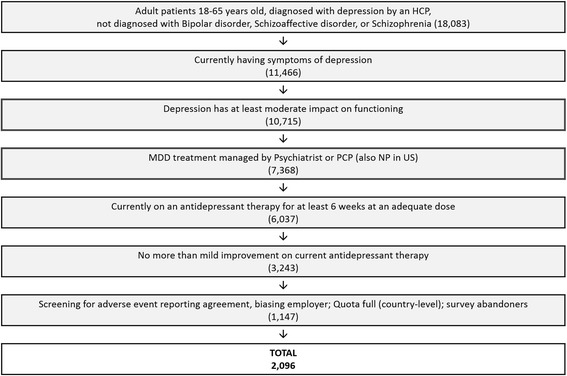
Screening disposition for participation in online patient survey

Current symptoms of depression were assessed using a modified version of the Patient Health Questionnaire (PHQ-9) [10], excluding the question about suicidal ideation. The scale was the standard PHQ-9 where 0 = Not at all, 1 = Several days, 2 = More than half of the days and 3 = Nearly every day. Other (non-PHQ-9) current symptoms were elicited from a check list of clinical symptoms associated with depression (see list in Fig. 1). The lists of emotional feelings regarding their treatment and HCP, included in the survey were created using direct patient input from qualitative interviews conducted during the survey development (not shown). Reasons for frustration were asked using a voluntary open-end format and responses were manually coded. Coded responses were validated by a second coder and quality checked by a research analyst. A list of consequences of frustration was also included in the survey based on patient and physician input during pre-survey qualitative interviews. To gauge the patient’s perception on the overall level of impairment caused by their depression, the survey included the additional PHQ item 10 “*How difficult have these problems made it for you to do your work, take care of things at home, or get along with other people?*”, where the scale included was 1 = Not difficult at all, 2 = Somewhat difficult 3 = Very difficult and 4 = Extremely difficult.

### Statistical analyses

Descriptive statistics included means and frequency of responses (%). A driver analyses was conducted using random forest analysis [11]. A random forest is a method where many decision trees (500 in our analysis) are constructed. The analysis selects a random sample of cases as the training set for each tree, and at each potential branching of the tree, a random subset of variables is evaluated as potential splits. The output of a random forest analysis provides ratio-level data of the influence that each characteristic has on the presence of dependent variable. Higher scores mean that, on average, the nodes created when the tree splits on this variable are more homogenous than nodes created by splitting on variables with lower scores – in other words they are considered more important. Patient characteristics entered as independent variables into the analysis included time since first episode, past hospitalization, past psychotherapy, number of episodes experienced, length of current episode, number of ADT failures, current treatment of monotherapy vs. combination therapy, change on current treatment (PGI-C), level of impairment (PHQ item 10) and disruption of daily living (SDS mean score). The dependent variable was the presence of moderate-to-severe frustration with their treatment. The random forest analysis was performed using the “randomForest” statistical package in R. Validity testing was done using a logistic regression to determine the explanatory power of the entered predictors.

## Results

### Respondent characteristics

A total of 18,083 adult (age 18–65) respondents diagnosed with MDD by a health care professional with no comorbid diagnoses of Bipolar Disorder, Schizoaffective or Schizophrenia were identified through our screening process using consumer panels (Fig. 1). Of the respondents with MDD, 11,466 were currently suffering from symptoms of depression and 93.5% of these (10,715) had at least a moderate impact on functioning. Approximately 56.3% (*n* = 6037) were being treated with an adequate dose of ADT for at least six weeks under the supervision of a psychiatrist, primary care physician (or nurse practitioner in the US). Of those adequately treated, 54% (3243) were experiencing an inadequate response to ADT. Finally, with drop-outs and quota caps at a country level, a total of 2096 respondents with inadequate response to ADT, completed the survey from six countries. These included the US (597, 28.5%), Canada (301, 14.4%), UK (300, 14.4%), Germany (299, 14.3%), France (299, 14.3%) and Spain (300, 14.4%).

The mean age of our survey respondents was 42.1 years and 56.7% were female (Table 1). While more than half of the respondents were working, 10% were on sick-leave or disability due to depression. The mean duration since onset of first episode was 10.5 years. Most respondents (91.1%) had suffered recurrent episodes and 39.6% had experienced over 10 episodes of depression. A third (34%) had been hospitalized for depression and 79% had received psychotherapy at some point.

**Table 1:** Demographic and clinical characteristics of respondents (*n* = 2096)

CHARACTERISTIC	*N* = 2096
Age; mean (years)	42.1
Gender; n (%) female	1188 (56.7)
Employment status; n (%)	
Working full/part time	923 (44.0)
Working, but currently on a sick leave/disability due to depression	219 (10.4)
Working, but currently on a sick leave/disability for another reason	80 (3.8)
Unemployed	308 (14.7)
Retired	184 (8.8)
Student	80 (3.8)
Homemaker	170 (8.1)
Other	132 (6.3)
Time since first episode; mean (years)	10.5
Number of total episodes; n (%) of respondents	
1	186 (8.9)
2–5	564 (26.9)
6–10	328 (15.6)
> 10	829 (39.6)
Don’t know	189 (9.0)
Recurrent episodes; n (%) of respondents	1721 (91.1)
Ever hospitalized for their depression; n (%) of respondents	713 (34.0)
Ever had psychotherapy; n (%) of respondents	1655 (79.0)
Duration of current episode; n (%) of respondents	
0–6 months	766 (36.5)
7–12 months	319 (15.2)
2–5 years*	457 (21.8)
> 5 years*	554 (26.4)
Patients receiving two or more classes of ADT; n (%) of respondents	799 (38.1)
Patients receiving adjunctive antipsychotic**; n (%) of respondents	345 (16.4)
Number of ADT failures in current episode; n (%) of respondents	
1	361 (17.2)
2	722 (34.4)
3+	1013 (48.3)

*Patients may have difficulties remembering symptom-free periods in between 2 episodes, leading to report of long lasting single episodes

**includes both atypical and typical antipsychotics

Per the screening requirements, all respondents were suffering from a current episode of depression. Just over half (51.7%) had been experiencing their current episode of depression for a year or less. Just over a third (38.1%) were taking combination ADT (at least two classes), and 16.4% were taking an adjunctive antipsychotic (AP).

Despite ongoing AD treatment, nearly a quarter of patients (23.7%) indicated their depression had gotten worse since the onset of their current episode, while another 20.4% indicated no change, 48.7% indicated minimal improvement, and only 7.2% indicated moderate-great improvement (PGI-C). The SDS mean score was 6.9, with mean domain scores of 7.5, 6.9 and 6.5 in work/school, social life, and family life/home responsibilities, respectively. Respondents’ rated their overall level of impairment caused by depression as a mean of 2.8, with 60.6% indicating ratings of very or extremely difficult (PHQ item 10). Analysis of individual PHQ-9 items revealed that more than two-thirds of patients reported feeling down, depressed or hopeless, or having little interest or pleasure in doing things more than half the days (33.3 and 30.6%, respectively) or nearly every day (36.5 and 38.0%) (Additional file 1: Appendix). The most frequently reported ongoing symptoms other than those measured by PHQ-9, was lack of motivation (77.2%) followed by anxiousness, irritability and excessive worries, (67.1%, 58.1 and 57.4%).

### Respondent emotions regarding their medication

**Fig. 2:**
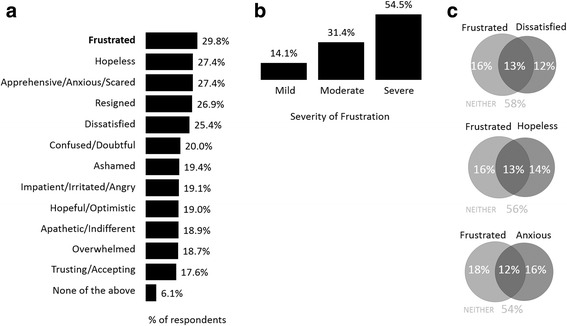
Respondent emotions regarding their medication (**a**) survey population (*n* = 2096), (**b**) severity of frustration when reported (*n* = 536), (**c**) overlap of frustration with hopelessness, anxiousness and dissatisfaction

Respondents were asked to describe how they felt about their prescription therapy and to select as many feelings that were relevant to their situation. Frustration was the emotion selected most often (29.8%) by respondents. Other emotions most often cited included, hopeless (27.4%), apprehensive/ nervous/anxious/scared (27.4%), resigned (26.9%), and dissatisfied (25.4%) (Fig. 2a). Of the respondents that indicated they were frustrated with their treatment, 85.9% rated their frustration as being moderate-to-severe (Fig. 2b). In addition, these respondents reported high levels of concomitant hopelessness (44.8% of those frustrated), anxiousness (48.2%) and dissatisfaction (44.8%) (Fig. 2c).

### Respondent emotions regarding their HCP

**Fig. 3:**
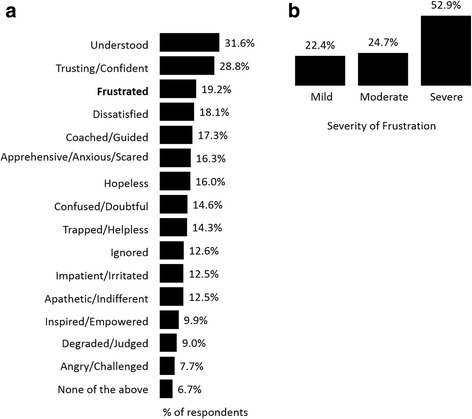
Respondent emotions regarding their HCP (**a**) survey population (*n* = 2096), (**b**) severity of frustration when reported (*n* = 311)

Patients with an inadequate response to their ADT were also asked how they felt about their HCP by selecting as many feelings that were relevant to their situation. The two most frequently chosen emotions were ‘feeling understood’ (31.6%) and ‘trusting/confident’ (28.8%). However, 19.2% of respondents reported frustration (Fig. 3a). Of those indicating they were frustrated with their HCP, 78% rated their frustration as being moderate-to-severe (Fig. 3b). Finally, of the 38.0% of patients who reported being frustrated with medication and/or their HCP, nearly a third reported frustration with both medication and their HCP (10.9% of all respondents).

### Reasons for feeling frustrated

**Fig. 4:**
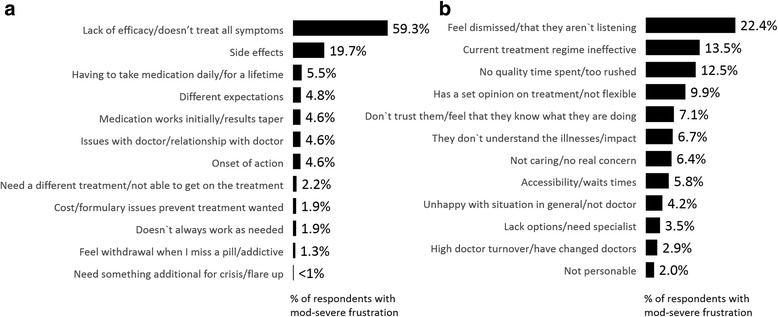
Reasons for frustration with (**a**) medication (*n* = 536) and (**b**) HCP (*n* = 311)

Respondents who indicated they were moderately-to-severely frustrated with either their treatment and/or their HCP were asked why they were frustrated via an open-end question format. Responses were then coded for select themes and are shown in Fig. 4. The top reason for their frustration with medication was lack of efficacy (59.3%) followed by side-effect issues (19.7%). Other reasons included having to take medication daily, failing to meet their expectations, tapering effect, their relationship with their physician and onset of action (Fig. 4). Frustration with their HCP was driven by respondents feeling dismissed/HCP is not listening to them (22.4%), current treatment not being effective (13.5%) and lack of quality time spent with patient/too rushed (12.5%) among other reasons shown in Fig. 4b.

### Clinical characteristics associated with higher frequency of frustration

**Fig. 5:**
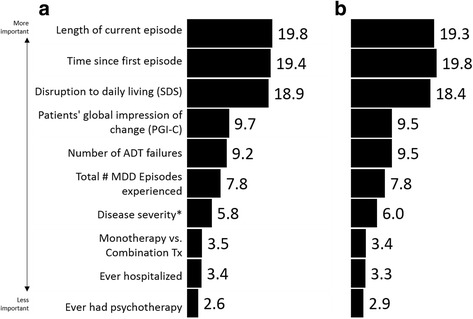
Relative contribution of respondent characteristics on the presence of frustration with (**a**) medication (**b**) their HCP

Random forest analyses indicated that for both frustration with medication and frustration with HCP the three largest drivers of frustration, of those entered in the model, were length of current episode, time since first episode and impact on function (SDS). Further, these three were twice as important as PGI-C and number of treatment failures (Fig. 5). The validity results however yielded a very small effect (Nagelkerke R-square = 0.074 for medication and 0.087 for HCP).

### Consequences/impact of feeling frustrated

**Fig. 6:**
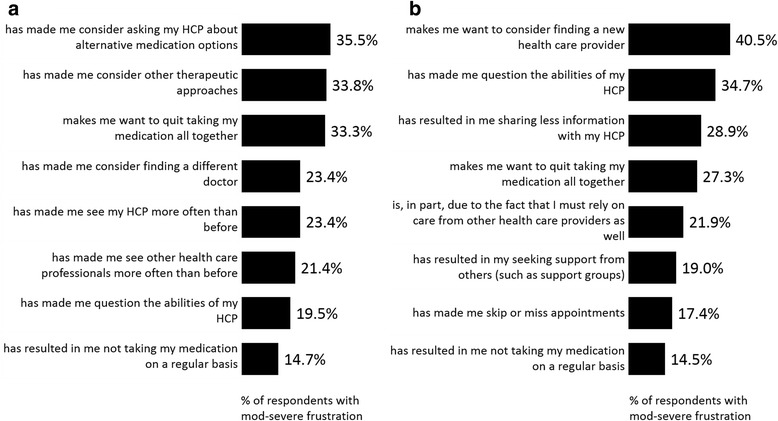
Consequences of frustration with (**a**) medication (*n* = 536) and (**b**) HCP (*n* = 311)

Patients moderately-to-severely frustrated with their treatment for depression were asked to identify how their frustration impacts their behaviors and decisions around their depression. The most frequently cited outcomes of frustration include patients considering alternative medication options (35.5%), other therapeutic approaches (33.8%) or simply quitting their medication all together (33.3%) (Fig. 6a). Another 14.7% admit that their frustration has resulted in them not taking their medication on a regular basis (Fig. 6a).

Those frustrated with their HCP suggested the frustration made them want to find a new HCP (40.5%), question the ability of their HCP (34.7%), share less information with their HCP (28.9%) and quit their medication altogether (27.3%) (Fig. 6b).

## Discussion

In line with the STAR*D study [3], our screening process demonstrates that just over half (54%) of all patients being treated for an ongoing episode of depression experience an inadequate response, and that this is associated with significant impairment in functioning (mean SDS score of 6.9). Beyond the presence of unresolved symptoms and impaired functioning, the results of this patient survey further suggest that an inadequate response to antidepressants may have other consequences that are important to consider as a healthcare provider.

It has been suggested that protracted depressive illness and treatment failure can lead to the erosion of critical social support, along with increased interpersonal, vocational and economic stress [12]. Studies have shown that having an inadequate response increases the duration of MDD and imposes a significant burden to society in terms of healthcare costs and workplace productivity losses [7]. This survey further supports the critical importance of addressing inadequate response in a timely and effective manner. Our results suggest that an inadequate response to antidepressants is often associated with negative emotions towards medication and HCP, including feelings of frustration, hopelessness and dissatisfaction. Frustration was the most frequently cited emotion regarding medication, with two in five patients indicating their frustration. The primary reasons for frustration with medication were directly related to the unresolved symptoms and side-effects of ADTs. A major finding of STAR*D was that likelihood of responding to a third or fourth step treatment after failing the initial two steps was < 20% [3]. Thus, the high levels of frustration with medication may reflect the fact that current treatment approaches/medications have not been effective, and that there is still an unmet need for treatments with an improved efficacy-tolerability profile.

In regards to HCPs, the most frequent emotions reported by respondents included two positive feelings of being understood, (31.6%) and confidence (28.8%), but the third most frequently cited emotion was frustration (19.2%). Interesting insight was gleaned from respondents when asked why they were frustrated with the HCP. Along with citing lack of response/ineffective treatment, patients identified dynamics of visit and relationship with HCPs. In fact, two of the top three reasons cited included feeling like the HCP is not listening to them/dismissing them and feeling like the visit is rushed and does not allow for quality time/discussion. This study also suggests that a patient who is frustrated with their medication is 4 times more likely to be frustrated with their HCP than those not frustrated with medication, underscoring the importance of addressing inadequate response to medication to assure good relations/collaboration.

Evaluation of the drivers of frustration using a random forest model showed that length of time since first episode, length of current episode and impact on functioning were the three highest predictors/drivers of frustration, both with medication and with HCP. However, it is important to remember that our explanation power, or lift in this case, is low suggesting there are many things influencing feelings of frustration that are not included in the model. For example, we observed that patients in France were less likely to report frustration with medication or HCPs than patients in the US, UK and Canada (data not shown). This may be due to cultural differences or differences in the healthcare system, but we were unable to explore this interesting observation within the limits of the survey design.

The results of this survey suggest that recognizing that a patient is frustrated with his/her medication is important for a timely intervention to reduce the negative impact of this frustration on adherence to treatment. Overall, 15% and 17% of respondents who reported being moderately-to-severely frustrated with their medication or HCP, respectively, said they have already stopped taking their medication on a regular basis. Another 33% and 27% indicated that their frustration with their medication and HCP, respectively, makes them want to “quit medication all together”. Moreover, nearly a third (29%) of patients frustrated with their HCP admitted to sharing less information, which may be critical to a treatment approach with HCP. Thus, frustration may represent a warning sign that a change in treatment is warranted. Asking patients to talk about their feelings about treatment and engaging patients on communicating their frustration concretely may help strengthen the patient/physician relationship and improve patient outcomes.

To the best of our knowledge, it is the first survey to evaluate the impact of frustration in patients with MDD. Strengths of this survey include its size and international approach. While this allowed us to note some interesting differences between countries, we did not explore the influence of culture or differing healthcare systems on the feelings of frustration. For example, we did not include type of healthcare (or type of healthcare provider) in the random forest analysis. The survey was conducted in Western countries, which limits its generalizability to other regions of the world. Other limitations include those inherent to patient self-report surveys, which are based on the patient’s own understanding of their condition, and are not compared with objective clinical information (e.g. about symptom severity, or response to treatment). Indeed, since our intent was to examine the patient perspective, we purposefully used subjective scales (instead of more standard clinical scales) to define an ‘inadequate response’ (PGI-C) and level of impairment (PHQ-9 item 10). Patients were recruited from existing consumer panels, and we do not know how this may have biased the results. For example, it may be that patients who are frustrated with their healthcare are more likely to respond to health surveys. Conversely, they could be less motivated to reply to the invitation.

In summary, this survey findings suggest that patients with prolonged disease (total or current episode), multiple treatment failures and impact on daily living may lead to feelings of frustration. In turn this frustration can lead to destructive behaviors such as non-compliance, mistrust of the HCP and patient’s withholding information that may be relevant to treatment decisions.

## Conclusions

Patients experiencing an inadequate response to treatment not only have unresolved depressive symptoms but also often have negative feelings about their failed treatments. This survey suggests that inadequate response to ADTs and continued trial-and-error of treatments can result in frustration, dissatisfaction, and other negative feelings towards medication and HCP. Since feelings of frustration may lead to adherence issues it is important that health care providers realize the emotional consequences of inadequate treatment and strive to reduce the levels of inadequate responses by exploring alternative treatment options.

## Additional file

Additional file 1: Appendix. Table e1. Antidepressants taken by respondents. **Figure e1**. Symptom frequency (a) individual PHQ-9 items (b) symptoms not assessed by PHQ-9. (PDF 71 kb)

## Abbreviations

ADT: Antidepressant therapy
HCP: Healthcare professional
MDD: Major depressive disorder
