# Supplementary material for: Understanding the emotions of patients with inadequate response to antidepressant treatments: results of an international online survey in patients with major depressive disorder
Source: BMC Psychiatry. 2018 Feb 5;18:33. doi: 10.1186/s12888-018-1625-y (PMC5800100; doi:10.1186/s12888-018-1625-y)

Appendix:

Table e1. Antidepressants taken by respondents

% of patients on…	Minimal dose (mg)	Total N=2,096	US n=597	Canada n=301	UK n=300	Germany n=299	France n=299	Spain n=300
**SSRI**		76.7%	69.8%	78.1%	70.3%	72.2%	87.3%	89.0%
citalopram	20							
escitalopram	10							
fluoxetine	20							
fluvoxamine	100							
paroxetine	20							
paroxetine CR	37.5							
sertraline	100							
**SNRI**		33.8%	22.4%	41.5%	21.0%	39.1%	40.1%	49.7%
desvenlafaxine	50							
duloxetine	60							
levomilnacipran	40							
milnacipran	100							
venlafaxine	75							
**MAOI**		6.8%	0.2%	9.3%	2.7%	14.4%	14.0%	0.0%
phenelzine	60							
tranylcypromine	30							
**TCA**		18.8%	5.2%	17.3%	21.7%	26.8%	25.8%	29.3%
amitriptyline	75							
clomipramine	100							
desipramine	100							
imipramine	75							
nortriptyline	75							
**OTHER ADTs**		24.2%	23.8%	25.6%	20.3%	31.1%	18.1%	27.0%
agomelatine	25							
bupropion	200							
mirtazapine	30							
trazodone	150							
vilazodone	20							
vortioxetine	20							

Figure e1. Symptom frequency (a) individual PHQ-9 items (b) symptoms not assessed by PHQ-9 (n=2,096)

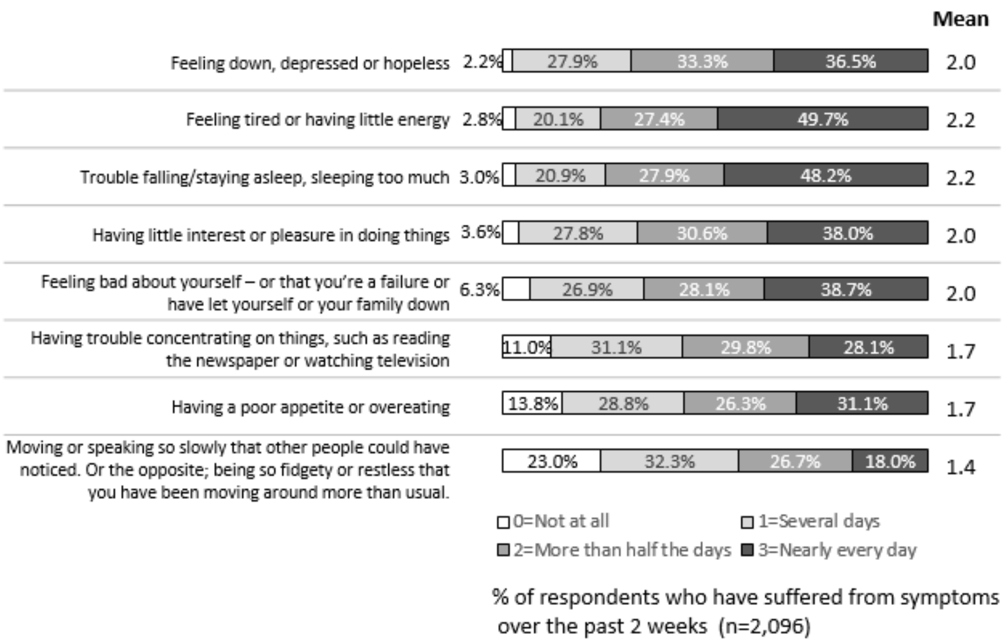

Supplement: Additional file 1: Appendix. Table e1. — Antidepressants taken by respondents. Figure e1. Symptom frequency (a) individual PHQ-9 items (b) symptoms not assessed by PHQ-9. (PDF 71 kb) [file 12888_2018_1625_MOESM1_ESM.pdf]
